# QTL fine-mapping of soybean (*Glycine max* L.) leaf type associated traits in two RILs populations

**DOI:** 10.1186/s12864-019-5610-8

**Published:** 2019-04-02

**Authors:** Liang Wang, Yanbo Cheng, Qibin Ma, Yinghui Mu, Zhifeng Huang, Qiuju Xia, Gengyun Zhang, Hai Nian

**Affiliations:** 1 The State Key Laboratory for Conservation and Utilization of Subtropical Agro-bioresources South China Agricultural University, Guangzhou, 510642 Guangdong People’s Republic of China; 2 The Key Laboratory of Plant Molecular Breeding of Guangdong Province, College of Agriculture, South China Agricultural University, Guangzhou, 510642 Guangdong People’s Republic of China; 3 The Guangdong Subcenter of the National Center for Soybean Improvement, College of Agriculture, South China Agricultural University, Guangzhou, 510642 Guangdong People’s Republic of China; 4 Beijing Genomics Institute (BGI)-Shenzhen, Shenzhen, 518086 People’s Republic of China

**Keywords:** Soybean, Leaf type traits, QTL, Fine mapping

## Abstract

**Background:**

The different leaf type associated traits of soybean (*Glycine max* L.) including leaf area, leaf length, leaf width, leaf shape and petiole length are considered to be associated with seed yield. In order to identify quantitative trait loci (QTLs) affecting leaf type traits, two advanced recombinant inbred line (RIL, ZH, Zhonghuang 24 × Huaxia 3; GB, Guizao 1 × Brazil 13) populations were introduced to score phenotypic values in plants across nine different environments (years, seasons, locations and soybean growth stages). Two restriction site-associated DNA sequencing (RAD-seq) based high-density genetic linkage maps with an average distance of 1.00 centimorgan (cM) between adjacent bin markers were utilized for QTL fine mapping.

**Results:**

Correlation analysis showed that most of the traits were correlated with each other and regulated both by hereditary and environmental factors. A total of 190 QTLs were identified for leaf type associated traits in the two populations, of which 14 loci were found to be environmentally stable. Moreover, these detected QTLs were categorized into 34 QTL hotspots, and four important QTL hotspots with phenotypic variance ranging from 3.89–23.13% were highlighted. Furthermore, *Glyma04g05840*, *Glyma19g37820*, *Glyma14g07140* and *Glyma19g39340* were predicted in the intervals of the stable loci and important QTL hotspots for leaf type traits by adopting Gene Ontology (GO) enrichment analysis.

**Conclusions:**

Our findings of the QTLs and the putative genes will be beneficial to gain new insights into the genetic basis for soybean leaf type traits and may further accelerate the breeding process for reasonable leaf type soybean.

**Electronic supplementary material:**

The online version of this article (10.1186/s12864-019-5610-8) contains supplementary material, which is available to authorized users.

## Background

Soybean (*Glycine max* L.) is one of the major crops abundant in protein and oil contents, which can fix nitrogen via microorganisms in the soil as well as a model plant for legume research [1, 2]. Reasonable leaf type traits are essential for soybean yield improving. Heath and Gregory first emphasized the strong relationships between leaf area (LA) and yield [3]. Decades later, Board and Harville demonstrated that LAI values required at least 3.5–4.0 in early reproductive growth stages for the maximum yield in soybean [4]. Likewise, leaf length (LL), leaf width (LW) and leaf shape (LS) have also been widely focused by breeders for many years [5–9]. Noteworthily, petiole length (PL) also makes much sense to soybean yield. According to the study of Jun and Kang, a short petiole length phenotype was more favorable for altering plant leaf angles to a vertical inclination form and improving plant density to obtain a high seed yield [10]. Therefore, it is imperative to dissect the genetic basis of these leaf type traits in soybean breeding.

With the development of next generation sequencing (NGS), discovering and genotyping for high-density single nucleotide polymorphisms (SNPs) data throughout the whole genome is now possible, which might useful to gain more information for marker-assisted selection (MAS) breeding process [11]. Genetic maps are effective tools for finding, dissecting and modifying the genes that determine simple and sophisticated traits in crops [12]. Restriction-site association DNA (RAD) technique together with the NGS is a cost-effective method that can simultaneously detect thousands of SNPs [13], and has been used for integrating high-density linkage maps and genetic analysis in plants, such as rice [14], sunflower [15], wheat [16], grape [17] and soybean [18–21]. Recently, genome-wide association study (GWAS) has become popular due to its high-resolution mapping when it was compared to conventional linkage mapping for dissecting complex genetic plant traits [22]. For instance, Li et al. genotyped 245 sorghum accessions by 85,585 SNPs and a total of 42 SNPs were identified to be associated with the five forage quality-related traits [23]. In another study, Phan et al. developed a core collection of 192 tomato accessions and evaluated for six fruit traits. As a result, they identified two loci for fruit color, seven loci for fruit shape, 11 loci for pericarp thickness, 13 loci for fruit weight, seven loci for fruit height, and ten loci for fruit width [24]. Fang et al. collected 809 soybean accessions worldwide and phenotyped them for 84 agronomic traits, 245 significant genetic loci for the target traits were discovered through GWAS [25].

A number of important bi-parental and GWAS QTLs for soybean agronomic traits have been reported over decades. To date, at least 40, 68, 66 and 87 QTLs controlling LA, LL, LW and LS have been detected, respectively (https://www.soybase.org/), based on various hereditary backgrounds, environments and statistical methods. Moreover, there is no QTL record for PL on SoybaseDatabase. In this study, we focused on LA, LL, LW, LS and PL across multiple environments by using two RIL populations as well as their RAD-seq based high-density genetic maps. The research aims of our study are as follows: (1) to map QTLs for leaf type associated traits in RIL populations and compare these data with previous research on SoybaseDatabase, (2) to determine if any QTLs were stable across multiple environments, (3) to select candidate genes in QTLs-based genetic intervals using Gene Ontology (GO) enrichment analysis.

## Results

### Phenotypic analysis of RIL populations

**Fig. 1:**
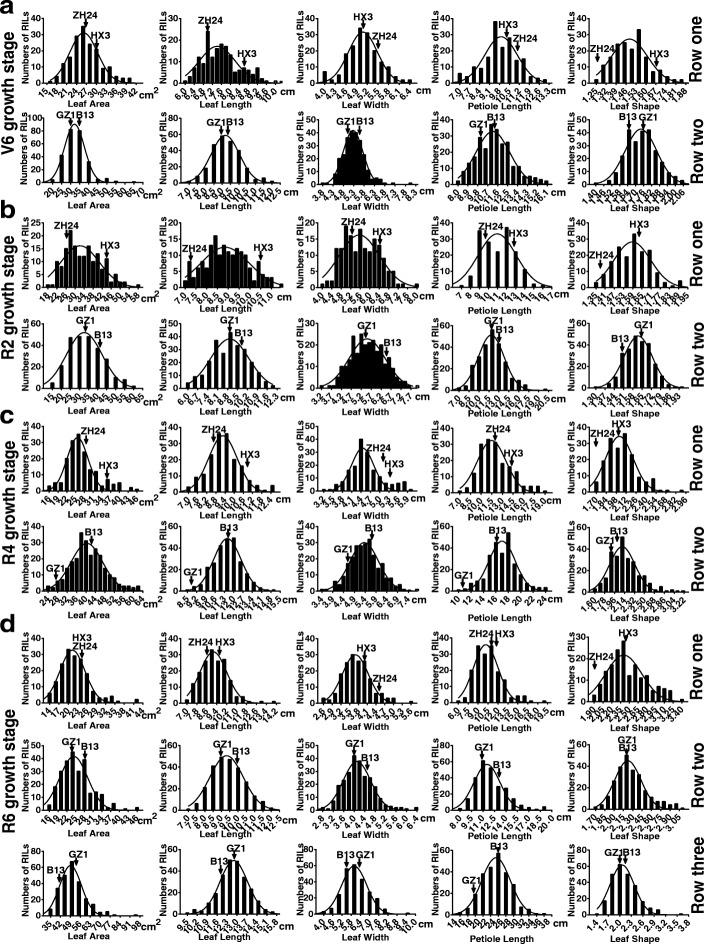
Frequency distributions for leaf type associated traits in ZH and GB RILs. The arrows indicate traits related values for the two parents used to construct the RIL population (cv. Zhonghuang 24 and Huaxia 3; cv. Guizao 1 and Brazil 13). **a** The frequency distribution for leaf type associated traits at soybean V6 growth stage, the ‘Row one’ and ‘Row two’ are corresponding to ZH and GB RILs in the summer of 2017 at Zengcheng, respectively; **b** The frequency distribution for leaf type associated traits at soybean R2 growth stage, the ‘Row one’ and ‘Row two’ are corresponding to ZH and GB RILs in the spring of 2017 at Zengcheng, respectively; **c** The frequency distribution for leaf type associated traits at soybean R4 growth stage, the ‘Row one’ is corresponding to ZH RILs in the summer of 2017 at Zengcheng, the ‘Row two’ is corresponding to GB RILs in the spring of 2016 at Zengcheng; **d** the frequency distribution for leaf type associated traits at soybean R6 growth stage, the ‘Row one’ and ‘Row two’ are corresponding to ZH and GB RILs in the summer of 2017 at Zengcheng, respectively; the ‘Row three’ is corresponding to GB RILs in the summer of 2017 at the Guangzhou experimental station

Phenotypic values for each RILs of two populations were performed across multiple environments. In most cases, leaf type traits of the male parent soybeans (‘Huaxia 3’ and ‘Brazil 13’) took higher values compared with those of female parents (‘Zhonghuang 24’ and ‘Guizao 1’), providing ideal materials for map-based QTL analysis. Moreover, the phenotypic values floated with different environments. Overall, the RIL phenotypic values presented wide spans and displayed continuous distributions. The frequency distributions of individual phenotypic data for two RIL populations were depicted in Fig. 1. As is shown in the figure, the segregations of these traits fit skew normal or normal distribution models, with typical quantitative genetic characteristics. Furthermore, the skewness and kurtosis of the distributions were listed in Tables 1 and 2. Notably, there were transgressive segregations both found in two RILs suggesting the existence of positive-effected alleles in the parental soybeans.

**Table 1:** Leaf type traits in ZH RILs in different environments

Traits	Parents Average		RILs Lines Average				Skewness	Kurtosis	Year, seasons, location and growth stages
	ZH24^a, b^	HX3^a, b^	Minimum^a, b^	Maximum^a, b^	Mean^a, b^	SD^a, b^			
LA^a^	26.85 ± 3.43	30.02 ± 4.88	16.86	40.96	26.85	4.77	0.49	0.09	2017Sum-Z-V6
LL^b^	6.91 ± 0.43	8.57 ± 0.78	6.04	9.98	7.60	0.82	0.54	−0.19	
LW^b^	5.50 ± 0.36	5.10 ± 0.42	3.91	6.36	5.09	0.47	0.04	0.23	
PL^b^	11.12 ± 1.89	10.39 ± 1.56	6.80	12.97	9.84	1.24	−0.14	0.01	
LS	1.26 ± 0.03	1.68 ± 0.06	1.26	1.86	1.49	0.12	0.48	0.09	
LA^a^	26.42 ± 2.21	44.18 ± 3.65	18.68	57.24	33.67	7.68	0.50	−0.27	2017Spr-Z-R2
LL^b^	7.25 ± 0.37	10.46 ± 0.51	7.02	11.28	8.92	0.97	0.17	−0.56	
LW^b^	5.25 ± 0.22	6.43 ± 0.26	4.06	7.83	5.63	0.75	0.28	− 0.53	
PL^b^	9.73 ± 0.73	12.79 ± 0.82	6.98	16.11	11.02	1.77	0.17	−0.75	
LS	1.38 ± 0.04	1.63 ± 0.03	1.36	1.92	1.59	0.11	0.35	0.15	
LA^a^	28.51 ± 3.14	35.86 ± 2.22	15.20	45.52	27.05	5.48	0.92	1.21	2017Sum-Z-R4
LL^b^	8.66 ± 0.50	10.85 ± 0.21	6.91	12.46	9.36	1.06	0.44	0.65	
LW^b^	5.13 ± 0.31	5.31 ± 0.29	3.21	5.91	4.55	0.53	0.58	0.43	
PL^b^	11.92 ± 1.43	14.35 ± 1.92	7.29	19.14	12.11	2.27	0.71	0.35	
LS	1.69 ± 0.06	2.04 ± 0.09	1.67	2.93	2.07	0.21	0.92	1.28	
LA^a^	24.44 ± 3.96	24.45 ± 3.82	13.50	42.99	22.23	4.91	1.23	2.83	2017Sum-Z-R6
LL^b^	8.61 ± 0.75	9.59 ± 0.80	7.04	13.84	9.21	1.11	0.70	1.50	
LW^b^	4.52 ± 0.46	3.94 ± 0.29	2.61	5.58	3.74	0.49	0.56	1.05	
PL^b^	10.96 ± 1.84	11.51 ± 1.21	6.41	18.94	10.56	2.14	0.93	1.27	
LS	1.91 ± 0.16	2.43 ± 0.13	1.86	3.45	2.49	0.33	0.59	−0.29	

*LA* leaf area, *LL* leaf length, *LW* leaf width, *PL* petiole length, *LS* leaf shape, *SD* Standard deviation;^a^ cm^2^, ^b^ cm, *2017Spr* 2017spring, *2017Sum* 2017summer, *Z* Zengcheng, *V6* V6 growth stage, *R2* R2 growth stage, *R4* R4 growth stage, *R6* R6 growth stage

**Table 2:** Leaf traits in GB RILs in different environments

Traits	Parents		RILs Lines				Skewness	Kurtosis	Years, seasons, locations and growth stages
	GZ1^a, b^	B13^a, b^	Minimum^a, b^	Maximum^a, b^	Mean^a, b^	SD^a, b^			
LA^a^	30.42 ± 4.74	34.72 ± 2.67	18.26	64.20	32.84	6.18	1.12	3.42	2017Sum-Z-V6
LL^b^	8.87 ± 0.80	9.26 ± 0.45	6.93	12.00	9.26	0.90	0.36	0.39	
LW^b^	5.03 ± 0.39	5.60 ± 0.22	3.85	8.17	5.31	0.55	0.86	3.00	
PL^b^	10.16 ± 1.20	11.38 ± 0.74	8.07	16.19	11.35	1.60	0.50	0.19	
LS	1.76 ± 0.03	1.65 ± 0.06	1.41	2.07	1.75	0.11	0.19	−0.21	
LA^a^	32.85 ± 1.02	41.57 ± 2.09	13.51	59.74	33.73	9.25	0.35	−0.34	2017Spr-Z-R2
LL^b^	8.91 ± 0.18	9.74 ± 0.32	6.10	11.88	8.93	1.26	−0.07	− 0.49	
LW^b^	5.43 ± 0.10	6.44 ± 0.11	3.29	7.76	5.52	0.85	0.14	−0.43	
PL^b^	11.80 ± 1.03	13.01 ± 0.51	6.66	20.06	11.98	2.04	0.34	0.60	
LS	1.64 ± 0.04	1.51 ± 0.03	1.31	1.94	1.62	0.10	0.06	−0.03	
LA^a^	27.41 ± 4.69	42.64 ± 6.84	23.16	62.92	41.13	7.32	0.38	0.38	2016Sum-Z-R4
LL^b^	8.79 ± 0.94	11.55 ± 0.58	8.49	15.06	11.52	1.06	0.08	0.20	
LW^b^	4.60 ± 0.49	5.72 ± 0.71	3.34	7.66	5.40	0.70	0.17	0.16	
PL^b^	10.74 ± 1.43	16.18 ± 1.39	9.72	23.75	17.01	2.40	0.09	0.49	
LS	1.91 ± 0.20	2.02 ± 0.23	1.56	3.19	2.16	0.27	0.87	1.24	
LA^a^	23.54 ± 3.44	28.23 ± 4.53	15.86	46.38	25.30	5.24	0.90	1.39	2017Sum-Z-R6
LL^b^	8.90 ± 0.73	9.90 ± 0.87	7.04	12.09	9.39	0.96	0.30	−0.32	
LW^b^	4.04 ± 0.36	4.49 ± 0.42	2.78	6.28	4.17	0.58	0.69	1.02	
PL^b^	10.74 ± 0.91	13.13 ± 2.02	7.97	19.47	12.07	2.02	1.02	1.43	
LS	2.20 ± 0.16	2.21 ± 0.16	1.73	3.11	2.28	0.26	0.76	0.58	
LA^a^	52.57 ± 5.63	41.90 ± 2.54	32.63	94.89	50.95	8.94	0.99	2.15	2017Sum-G-R6
LL^b^	12.80 ± 0.08	11.87 ± 0.53	9.67	15.61	12.76	1.05	−0.12	0.26	
LW^b^	6.33 ± 0.47	5.53 ± 0.25	3.92	9.67	6.11	0.86	0.40	0.49	
PL^b^	17.48 ± 2.74	23.92 ± 3.49	13.80	35.91	23.52	3.67	0.15	0.25	
LS	2.02 ± 0.14	2.15 ± 0.09	1.47	3.59	2.12	0.34	0.89	1.16	

*LA* leaf area, *LL* leaf length, *LW* leaf width, *PL* petiole length, *LS* leaf shape, *SD* Standard deviation; ^a^ cm^2^, ^b^ cm*2016Sum* 2016summer, *2017Spr* 2017spring, *2017Sum* 2017summer, *Z* Zengcheng, *G* Guangzhou, *V6* V6 growth stage, *R2* R2 growth stage, *R4* R4 growth stage, *R6* R6 growth stage

The phenotypic value correlation analysis of the two RILs in each specific environment (Additional file 1: Tables S1 and S2) showed that most of the leaf type traits were highly correlated to each other and exhibited statistically significant (*P* < 0.01). Generally, LA and LW presented the highest positive correlation coefficients in the two populations. In this study, LA usually showed negative correlation to LS and the correlation coefficients in ZH population were universally weaker than those in GB population. Comparatively, LL and LW shared substantially positive correlation with each other. Moreover, LS was derived from the ratio LL to LW and exposed substantial positive correlation to LL and negative to LW in most cases. Interestingly, PL was highly associated with LA, LL and LW, but did not show significant relation to LS in the two populations. Furthermore, we analyzed the correlations between different environments for each target leaf type trait in two RILs. As is shown in Additional file 1: Tables S3 and S4, for the same planting location (Zengcheng), the correlation coefficients of various environments were broadly found to be significantly positive. In ZH RIL population, for all the five leaf type traits, the R4 (full pod reproductive) growth stage in the summer of 2017 at Zengcheng displayed the strongest correlations to other environments. Correspondingly, the most prominent environment in GB population was the R6 (full seed reproductive) growth stage in the summer of 2017 at Zengcheng. Particularly, for the different cultivating sites, the analysis data of GB RIL population at the R6 growth stage in the summer of 2017 at the Guangzhou experimental station presented widely weak relationship to those at Zengcheng.

### Exploration of leaf type trait QTLs in two RIL populations

**Fig. 2:**
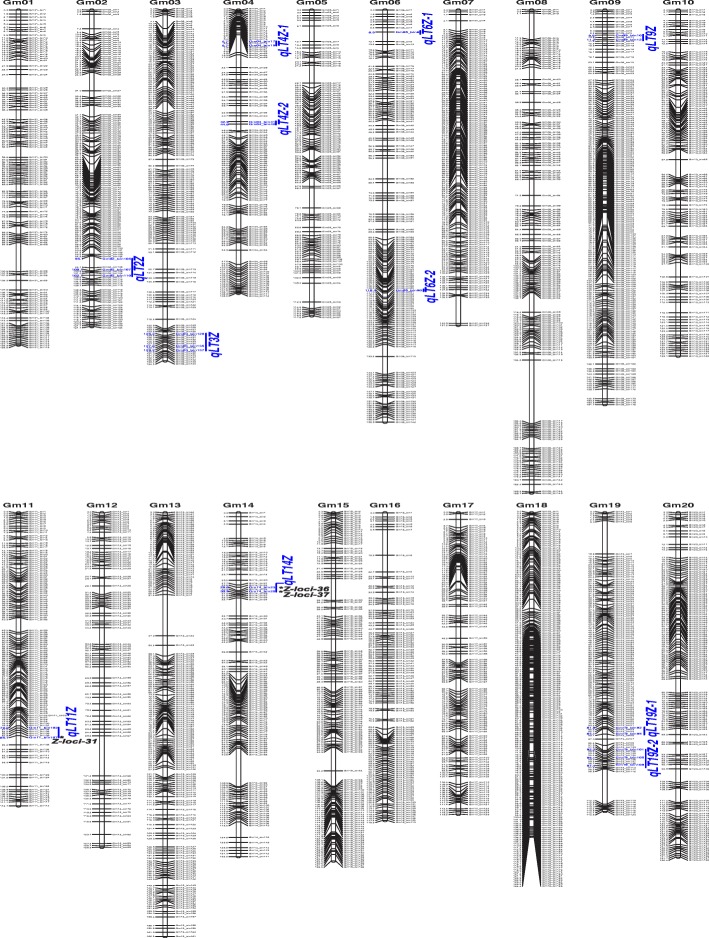
Soybean high-density genetic map of ZH RIL population. The bin markers and their locations are shown on the right and left sides, respectively. The three stable loci for leaf type associated traits are marked by asterisks in bold and the 11 QTL hotspots were highlighted in blue

**Fig. 3:**
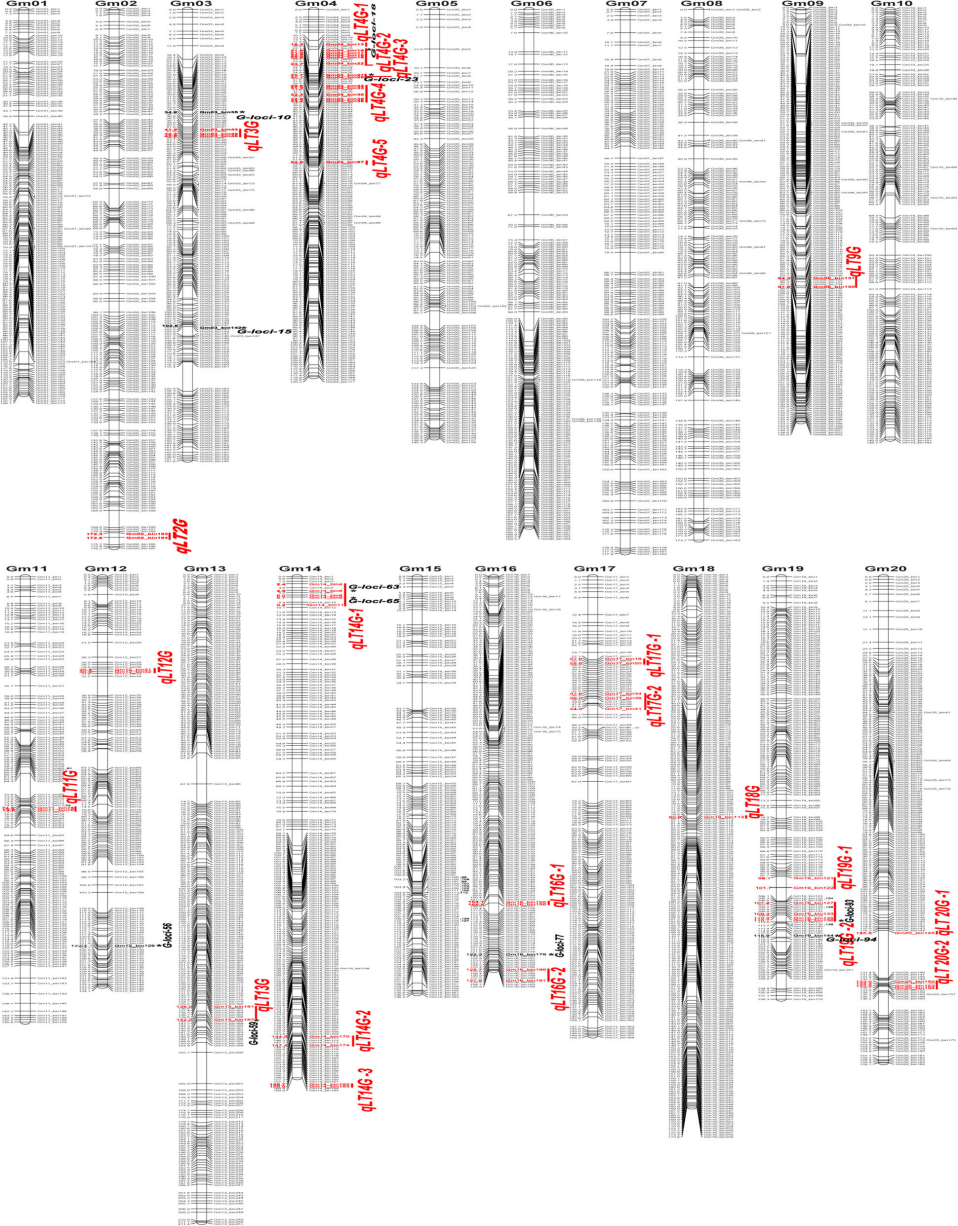
Soybean high-density genetic map of GB RIL population. The bin markers and their locations are shown on the right and left sides, respectively. The 11 stable loci for leaf type associated traits are marked by asterisks in bold and the 23 QTL hotspots were highlighted in red

With the SNP genotyping method, 47,472 and 56,561 high-quality polymorphic SNP sites were detected for ZH and GB RILs, respectively. All the SNP sites in the RILs were integrated as recombination bin units. As a result, 2639 recombinant bins were obtained for ZH RILs and 3715 bins for GB RILs (Additional file 1: Tables S5 and S6). Based on genotypes of these marker bins, two high-density bin linkage maps were constructed (Figs. 2 and 3), with an average distance of 1.00 cM between adjacent markers [20]. By taking the composite interval mapping (CIM) method as well as utilizing the corresponding maps, 190 QTLs for leaf type traits have been discovered in the two RIL populations.

In ZH RIL population, 56 QTLs for the target five leaf traits were identified on 14 chromosomes (02, 03, 04, 06, 08, 09, 10, 11, 12, 13, 14, 15, 17 and 19) (Additional file 1: Table S7). These QTLs were able to explain phenotypic variation ranging from 5.36% (*qLA14b*) to 18.23% (*qLW4b*) with the LOD values 2.51 to 7.86. Among these QTLs, 11, 10, 12, 11 and 12 QTLs were mapped for LA, LL, LW, LS and PL, respectively. *qLA4b* was detected by the bin19 marker on chromosome 4 in ZH population with phenotypic variance contribution up to 12.22% and was the most prominent QTL for LA. Moreover, *qLA4b* displayed a negative additive effect which indicated the positive functions of the alleles derived from ‘Huaxia 3’. Coincidentally, *qLW4b* was also identified by the bin19 marker which shared the physical interval 3,657,048 to 3,740,933 bp with *qLA4b* in the same environment and was the most dominant effect QTL for LW as well. Comparatively, *qLW4b* showed a greater phenotypic variance (*R*^2^ = 18.23%) and positive additive effect. Furthermore, we designated *qLL3b*, *qLS6a-2* and *qPL19d* as the leading QTLs for LL, LS and PL due to their relatively advanced genetic effects (*R*^2^ = 10.54, 10.92 and 15.70%).

Accordingly, there were 134 detected QTLs almost fully covered the whole genome (except for chromosome 15) through five circumstances in GB RIL population (Additional file 1: Table S8). Noticeably, a wider range phenotypic variance spanned from 2.71% (*qLS3i*) to 23.13% (*qLW4f-1*) and LOD score distributions from 2.56 to 16.15 were also demonstrated. An entire of 24, 26, 24, 34 and 26 QTLs were identified for LA, LL, LW, LS and PL in GB population. In addition, *qLA4f-1, qLL4f-1, qLW4f-1* and *qPL7g* were four major QTLs with the leading phenotypic variance contributions (*R*^2^ = 22.83, 22.53, 23.13 and 10.22%) in their respective traits. Moreover, all of them showed negative additive effects, which revealed the acquisition of favorable alleles from Brazil 13. Furthermore, *qLS19g-1, qLS19h* and *qLS19i-1* presented positive additive effects across three different environments and were three major QTLs (R^2^ ≥ 10%) for LS in GB population.

### Identification of stable loci and important QTL hotspots for leaf type traits

The entirely detected QTLs in ZH and GB RIL populations were then subdivided into 48 and 100 loci based on the overlapped bins (Additional file 1: Tables S7 and S8). The loci name was composited with the first letter of the RIL population name (known as ‘Z’ or ‘G’) and ‘loci’, then consecutively numbered with Arabic numerals. In the present study, there were 14 stable loci identified across multiple environments. In ZH RIL population, three loci (*Z-loci-31, Z-loci-36* and *Z-loci-37*) for three leaf type traits (LL, LA and LW) were mapped onto two soybean chromosomes (11 and 14). Interestingly, all of them showed negative additive effects. Correspondingly, there were 11 loci distributed on seven chromosomes (03, 04, 12, 13, 14, 16 and 19) in GB RILs, responding to all the five target leaf traits. Among them, six loci (*G-loci-10, G-loci-15, G-loci-59, G-loci-63, G-loci-77* and *G-loci-94*) were found to be positive additive effects and five (*G-loci-18, G-loci-23, G-loci-56, G-loci-65* and *G-loci-93*) presented negative effects. The details of them were listed in Table 3. Meanwhile, nearly all of them (except for *G-loci-93*) solely paralleled to one leaf type trait that may shed light on the genetic basis of specific leaf type trait construction.

**Table 3:** The 14 stable loci for leaf type traits in two populations across different environments

RILs name^a^	Loci name^b^	QTL name^c^	Chr^d^	Bin name	CI_v1.1_ (bp)^e^	Position (cM)	LOD^f^	ADD^g^	R^2^ (%)^h^	CI_v2.0_ (bp)^i^
ZH	*Z-loci-31*	*qLL11c-2*	11	Bin107	36,478,191–36,619,869	85.70	2.89	−0.33	7.10	32,011,930–32,147,322
		*qLL11d*	11	Bin107	36,478,191–36,619,869	85.70	2.99	−0.30	6.77	32,011,930–32,147,322
	*Z-loci-36*	*qLA14a*	14	Bin23	4,871,116–4,920,206	29.80	2.71	−1.18	6.06	4,954,181–5,005,224
		*qLA14c*	14	Bin23	4,871,116–4,920,206	29.80	2.64	−1.48	6.42	4,954,181–5,005,224
	*Z-loci-37*	*qLW14a*	14	Bin25	5,292,323–5,333,084	30.90	4.40	−0.15	10.44	5,403,689–5,448,621
		*qLW14c-1*	14	Bin25	5,292,323–5,333,084	30.90	4.52	−0.19	10.86	5,403,689–5,448,621
GB	*G-loci-10*	*qPL3g-1*	3	Bin35	4,763,728–5,164,283	34.90	3.65	0.56	5.19	4,566,741–5,065,688
		*qPL3i-2*	3	Bin35	4,763,728–5,164,283	34.90	2.87	0.75	4.28	4,566,741–5,065,688
	*G-loci-15*	*qLS3h*	3	Bin142	41,133,188–41,201,407	102.80	3.30	0.05	3.61	39,126,123–39,181,924
		*qLS3i*	3	Bin142	41,133,188–41,201,407	102.80	2.56	0.06	2.71	39,126,123–39,181,924
	*G-loci-18*	*qLL4e-1*	4	Bin16	4,020,745–4,048,162	21.60	5.11	−0.26	7.51	4,070,205–4,094,078
		*qLL4g-1*	4	Bin16	4,020,745–4,048,162	21.60	13.82	−0.47	18.12	4,070,205–4,094,078
		*qLL4i*	4	Bin16	4,020,745–4,048,162	21.60	11.13	−0.44	15.84	4,070,205–4,094,078
	*G-loci-23*	*qPL4f*	4	Bin27	4,764,737–4,814,980	25.70	2.77	−0.42	4.01	4,822,857–4,873,263
		*qPL4g*	4	Bin27	4,764,737–4,814,980	25.70	4.63	−0.63	6.47	4,822,857–4,873,263
	*G-loci-56*	*qLS12g*	12	Bin126	36,772,134–36,809,388	122.10	2.63	−0.05	3.00	36,730,431–36,766,381
		*qLS12h*	12	Bin126	36,772,134–36,809,388	122.10	3.48	−0.05	4.06	36,730,431–36,766,381
	*G-loci-59*	*qLW13g*	13	Bin187	33,303,067–33,385,748	142.20	2.60	0.13	3.32	34,514,243–34,601,384
		*qLW13h*	13	Bin187	33,303,067–33,385,748	142.20	2.84	0.11	3.47	34,514,243–34,601,384
	*G-loci-63*	*qLS14e*	14	Bin6	495,526–538,526	4.50	11.01	0.04	13.67	506,220–544,002
		*qLS14f*	14	Bin6	495,526–538,526	4.50	2.89	0.02	3.95	506,220–544,002
		*qLS14h*	14	Bin6	495,526–538,526	4.50	2.78	0.05	3.06	506,220–544,002
	*G-loci-65*	*qLW14h*	14	Bin9	664,663–746,688	6.70	4.73	−0.09	7.08	659,428–753,278
		*qLW14i-1*	14	Bin9	664,663–746,688	6.70	3.85	−0.20	4.91	659,428–753,278
	*G-loci-77*	*qLS16e*	16	Bin179	35,798,290–35,892,430	122.30	12.01	0.05	15.30	36,302,667–36,395,478
		*qLS16i*	16	Bin179	35,798,290–35,892,430	122.30	2.67	0.06	2.83	36,302,667–36,395,478
	*G-loci-93*	*qLA19g*	19	Bin136	45,183,988–45,457,039	110.70	3.16	−1.52	4.24	45,304,487–45,567,452
		*qLA19h-2*	19	Bin136	45,183,988–45,457,039	110.70	4.69	−1.37	6.67	45,304,487–45,567,452
		*qLA19i*	19	Bin136	45,183,988–45,457,039	110.70	2.65	−1.79	3.89	45,304,487–45,567,452
		*qLW19g*	19	Bin136	45,183,988–45,457,039	110.70	12.83	−0.30	18.31	45,304,487–45,567,452
		*qLW19h-2*	19	Bin136	45,183,988–45,457,039	110.70	9.64	−0.21	12.76	45,304,487–45,567,452
	*G-loci-94*	*qLS19g-2*	19	Bin144	46,060,014–46,274,131	115.90	9.44	0.10	13.77	46,167,548–46,388,153
		*qLS19i-2*	19	Bin144	46,060,014–46,274,131	115.90	8.19	0.11	11.03	46,167,548–46,388,153

^a^RIL name: ZH Zhonghuang 24 × Huaxia 3 RIL population; GB Guizao 1 × Brazil 13 RIL population

^b^Loci name is composited with the first letter of RIL population name known as ‘Z’ or ‘G’ and ‘loci’ following its former order

^c^The name of QTL is a composite of the leaf traits: leaf area (LA); leaf length (LL); leaf width (LW); petiole length (PL); leaf shape (LS); a: the V6 growth stage in the summer of 2017 at Zengcheng; c: the R4 growth stage in the summer of 2017 at Zengcheng;d: the R6 growth stage in the summer of 2017 at Zengcheng; e: the V6 growth stage in the summer of 2017 at Zengcheng; f: the R2 growth stage in the spring of 2017 at Zengcheng; g: the R4 growth stage in the summer of 2016 at Zengcheng; h: the R6 growth stage in the summer of 2017 at Zengcheng; i: the R6 growth stage in the summer of 2017 at the Guangzhou experimental station

^d^Chr refers to chromosome

^e^The physical position corresponding to the 95% confidence interval for the detected QTL based on Glyma.Wm82. a1. v1.1 gene model

^f^LOD indicates the logarithm of odds score

^g^Positive and negative values indicated additive effect by the alleles of parents, respectively

^h^R^2^ indicates the phenotypic variance explained by individual QTL

^i^The most proximal Glyma.Wm82.a2.v1 gene model physical intervals of the detected QTLs were transformed by focusing on the positions of the interval nearest 3′ and 5′ ending genes

**Fig. 4:**
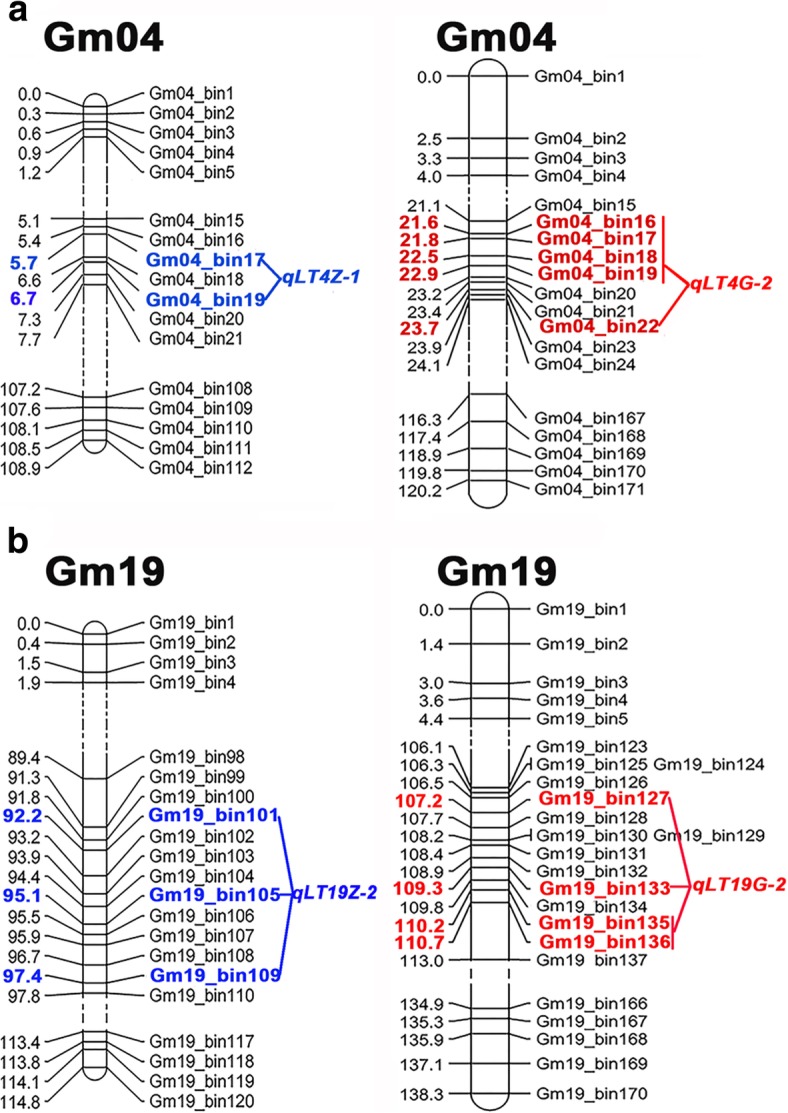
Important QTL hotspots of leaf type traits in ZH and GB RIL population. The virtual point lines represent the truncated segments of chromosomes. The QTL hotspot names are composites of leaf type traits (LT) followed by the chromosome number. The corresponding bin markers have been emphasized in bold. The QTL hotspots in ZH and GB RIL populations were colored in blue and red, respectively. **a** the two important QTL hotspots on chromosome 04; **b** the two important QTL hotspots on chromosome 19

Referring to Liu et al. determining criterion on QTL hotspots (so-called QTL clusters), the adjacent identified QTLs were categorized into 11 and 23 QTL hotspots for leaf type traits in ZH and GB RIL populations with a wide covering range on soybean genome [20]. Moreover, these QTL hotspots contained at least two QTLs and were named after ‘LT’ which represented the regulation of diverse leaf type traits. As is shown in Additional file 1: Table S9, the QTL hotspots of ZH RILs distributed on eight chromosomes (02, 03, 04, 06, 09, 11, 14 and 19). Compared to ZH RILs, the QTL hotspots of GB RILs spanned 13 chromosomes except for chromosomes 01, 05, 06, 07, 08, 10 and 15 (Additional file 1: Table S10). Intriguingly, most of the QTL hotspots with relatively high phenotypic variance contributions were converged into the genetic intervals on the front part of chromosome 04 and the posterior part of chromosome 19. Furthermore, four important stable QTL hotspots (*qLT4Z-1*, *qLT19Z-2*, *qLT4G-2* and *qLT19G-2*) were evenly located on chromosomes 04 and 19 for ZH and GB RIL populations across multiple environments (Fig. 4). In ZH population, *qLT4Z-1* was identified in an interval between 3,473,033 and 3,740,933 bp on chromosome 04 including three novel QTLs for three leaf type traits (LL, LA and LW) with the phenotypic variance up to 18.23% (*qLW4b*). Furthermore, *qLT19Z-2* was located in a genetic block 44,764,317 to 45,888,005 bp on chromosome 19 with the phenotypic contribution ascending to 15.7% (*qPL19d*) and is related to three QTLs for LW, PL and LS. In GB population, *qLT4G-2* was an entry new QTL hotspot across multiple environments, responding to the five target leaf traits and the highest phenotypic variance was 23.3% (*qLW4f-1*). Likewise, *qLT19G-2* with ten QTLs was able to explain the phenotypic variance ranging from 3.89% (*qLA19i*) to 20.53% (*qLS19i-1*) for LA, LW, PL and LS. Summarily, the four important QTL hotspots may play key role in regulating complicated traits in soybean leaves. Additionally, all of these stable loci and QTL hotspots have been marked on their accordant chromosomes in the constructed high-density genetic maps (Figs. 2 and 3).

### Gene ontology (GO) enrichment analysis and candidate gene prediction

In order to gain an in-depth understanding of which genes may relate to the integrated leaf type traits, we retrieved the gene calls in the genetic blocks of four important QTL hotspots. A total of 60 and 98 annotated genes were discovered in the genetic intervals of hotspots on chromosome 04 and chromosome 19, respectively (Additional file 1: Table S11). Among the 158 annotated genes, 81 were found to have at least one GO annotation. Furthermore, these genes were predicted to be related to various biological processes and could be grouped into 13 categories, including regulation of biological process, biological regulation, cellular process, metabolic process, establishment of localization, localization, transcription regulator activity, catalytic activity, binding, macromolecular complex, cell, cell part and organelle (Additional file 1: Table S12). Some biological processes such as metabolic processes, catalytic activity, and particularly transcription regulator activities are essential for gene expressions and metabolites in all organisms. Likewise, to further exploring the candidate genes of a certain specific leaf type trait, we focused on the 14 stable loci and carried out the analyzing procedures consistent with those on QTL hotspots. Subsequently, entire 206 gene calls were listed on (Additional file 1: Table S13) and GO analysis of the specific leaf type traits were summarized on (Additional file 1: Table S14).

By applying the GO enrichment analysis as well as considering the hereditary variation of mapping regions and the gene annotations on Phytozome (https://phytozome.jgi.doe.gov/pz/portal.html), we predicted four candidate genes. Among them, *Glyma04g05840* and *Glyma19g37820* were originated from the intervals of two important QTL hotspots (*qLT4G-2* and *qLT19Z-2*). Comparably, *Glyma14g07140* was found in the genetic region of a major stable loci (*Z-loci-37*) for LW in ZH RIL population, *Glyma19g39340* was originated from the hereditary interval of a major stable loci (*G-loci-94*) for LS in GB RIL population.

## Discussion

### Main effective factors for phenotype

Xavier et al. previously reported that complex traits in soybean are regulated and controlled by multi-genetic as well as environmental factors [26]. As is shown in Tables 1 and 2, the leaf type associated traits phenotypic data of ZH and GB RIL populations at Zengcheng floated with different growth stages. Moreover, for the specific target trait, the phenotypic data exhibited obvious differences in two RIL populations. Hence, we extrapolated that the leaf type traits may influenced both by the related gene time expressions and different hereditary backgrounds [27]. According to the correlation analysis in Additional file 1: Tables S1-S4, most of the leaf type traits highly interacted with each other and this may support that functional gene may be pleiotropic or closely linked to some extent [28]. Noticeably, due to the different planting sites, the phenotype data of the R6 growth stage in the summer of 2017 at the Guangzhou experimental station for GB RILs almost showed no strong correlation to those at Zengcheng. From this aspect, the leaf type traits may affect by the environmental factors in GB RILs. Generally, the results of present study showed relatively good consistency with the former research.

### Influencing factors for QTL mapping

Promoting genetic improvements of soybean by molecular approaches, which is one of the primary goals throughout soybean breeding [29]. The efficacy of QTL mapping is to acquire favorable alleles and seeking for genetic mechanisms. Many factors such as, parental hereditary diversity, environmental effects and molecular marker density may influence the precisions of QTL mapping [30]. The research aim of our study was to identify QTLs for five important leaf type traits in soybean. Previous studies have mapped different QTLs for these leaf type traits. Nevertheless, our study was distinct to the former researches in several important aspects. These included the application of two stably advanced RIL populations, which exceeded F_12_ generations as well as relatively abounding segregation lines (164 ZH RILs and 256 GB RILs). Furthermore, we utilized two RAD-seq based high-density genetic linkage maps, making the QTL mapping more accurate and reliable. Meanwhile, for the parental genetic variation term, the parent soybeans had at least two distinctly different leaf type traits in any circumstance (Tables 1 and 2). Comparatively, ‘Guizao 1’ and ‘Brazil 13’ had obvious differences in LA, LW and PL across most environments, and ‘Zhonghuang 24’ and ‘Huaxia 3’ were more significantly diversified in LL and LS. In addition, by adopting a scanning method, we improved the accuracy as well as the efficiency of phenotypic data collections. Notably, we aggregately analyzed the leaf traits in various environments throughout different seasons and growth stages. In brief, we attempted to find some constantly stable QTLs and provided the orientation for shifting leaf type traits as well as improving soybean breeding process.

### Comparisons of the detected QTLs in the current study and the previous research

Previously published QTLs for leaf type traits (LA, LL, LW and LS) widely distributed in the whole soybean genome [9, 25, 31–41] (Additional file 1: Table S15). In the present study, we compared and judged the relations between the detected leaf type QTLs in this study and the reported ones [9, 25, 31–35, 37–40] (Additional file 1: Tables S16 and S17). According to Additional file 1: Tables S16 and S17, the leaf type QTLs identified on chromosome 04 and chromosome 19 showed good inter-relevance between ZH and GB mapping populations. Notably, compared to the formerly published leaf type QTLs, most QTLs detected herein were novel ones. In addition, the detected QTLs in two populations on chromosome 19 also presented relatively strong correlations to the previously published leaf type QTLs. In comparison, on chromosome 04, the discovered genetic regions of the QTLs in current study have not been reported for QTL of leaf type trait before.

Interestingly, most of the detected QTLs which took considerable phenotypic variations in ZH and GB RIL populations were clustered into the hereditary regions on the front part of chromosome 04 and the posterior part of chromosome 19 (Additional file 1: Tables S7 and S8). In this study, we emphasized four important QTL hotspots in these regions. Many yield-related traits, like branching, pod number, seed weight, plant height, node number, seed set (so-called seed per pod) can affect soybean yielding [42–62]. *qLT19Z-2* and *qLT19G-2* are two important QTL hotspots correlated to leaf type traits identified in the genetic intervals on the posterior part of chromosome 19. As is shown in (Additional file 1: Table S18), a number of previously published QTLs for leaf type and yield-related traits clustered in the hereditary blocks of *qLT19Z-2* and *qLT19G-2* [25, 31, 34, 35, 38, 48–62]. Comparably, *qLT4Z-1* and *qLT4G-2* were novel QTL hotspots on chromosome 04 for leaf type traits in the current study. No early reported leaf trait QTL was identified in the genetic regions of the novel QTL hotspots. However, five and ten published yield-related QTLs were found in the hereditary blocks of *qLT4Z-1* and *qLT4G-2*, respectively [42–47]. In current study, the four important QTL hotspots contain 28 leaf type traits QTLs. The results in Additional file 1: Table S18 also demonstrated the correlations between the 28 leaf type QTLs and reported yield- related QTLs. Moreover, compared to former research, we fine mapped the QTLs for the petiole trait. Importantly, four detected QTLs for PL (*qPL19d*, *qPL4h-1*, *qPL4e-1* and *qPL19h*) were included by the four major leaf type QTL hotspots and corelated well with the previously reported and the present discovered QTLs. The coincidence of the QTLs across different genetic backgrounds and studies not only reveal the stability and reliability of the QTLs detected herein, but also highlight the significance of these regions in breeding to develop reasonable leaf type as well as high-yielding soybean cultivars.

### Four putative genes for soybean leaf type traits

Plant leaf and petiole developed from a group of cells named the leaf primordium, which initiated at the brink of the shoot apical meristem (SAM) [63, 64]. Subsequently, cell division, differentiation and expansion are temporally and spatially coordinated to convert the infant leaf into a mature leaf [64–66]. Many hormones like auxin and cytokinin have been supposed to participate in cell cycle processes [67–69]. Moreover, the plant cell metabolism is closely associated with the cell wall loosening ability [70, 71]. According to the (Additional file 1: Table S12), *Glyma04g05840* and *Glyma19g37820* have five and one GO annotations, respectively. *Glyma04g05840* contained a FAD domain and was noted the capability of binding both FAD and cytokinin substrates which participated in cytokinin metabolic process. Moreover, *Glyma04g05840* was derived from a major interval of *qLT4G-2* on chromosome 04 and was estimated relatively great expression in leaf tissues on Phytozome. Comparably, *Glyma19g37820* was obtained from the interval of *qLT19Z-2*. This gene contained a LysM domain with evaluatively significant expression in shoot tip and GO annotation showed its participation in cell wall macromolecule catabolic process which may be important in cell wall loosen ability adjustment. Likewise, *Glyma14g07140* and *Glyma19g39340* were originated from the hereditary interval of *Z-loci-37* and *G-loci-94*, respectively (Additional file 1: Table S13). Leaf width is influenced by the cell paraxial growth. In this study, *Glyma14g07140* had a HAT1 domain that considered to connect with the cell cycle and was noted relatively great expression in shoot apical meristem (SAM). Furthermore, it was identified from leaf width loci (*Z-loci-37*) in ZH RIL population. This gene may be valuable in controlling the cell paraxial growth in soybean leaf and affect leaf width. Another putative gene *Glyma19g39340* that encoded a B3 binding domain was an auxin response transcript factor gene, which was predicted to have a high expression in SAM as well. Taken together, we assumed these four genes as the candidate genes for soybean leaf type traits in this study. Nevertheless, these selected genes should be further probed in more prospective validations and comprehensively linked them to yield traits related genes to fully demonstrate their roles in soybean leaf development.

## Conclusions

In this study, we fine mapped five soybean leaf type associated traits by using two recombinant inbred line (RIL) populations (Zhonghuang 24 × Huaxia 3; Guizao 1 × Brazil 13) and their constructed high-density genetic maps. A total of 190 QTLs for leaf type associated traits were detected. Among them, 103 QTLs were found to be correlated to the published ones for leaf type traits. Moreover, 14 stable loci for specific leaf type trait were identified and four major QTL hotspots for relevant leaf type traits were classified. Furthermore, four candidate genes originating from the hereditary intervals of the stable loci and the important QTL hotspots were predicted. The putative genes may directly or indirectly affect soybean leaf type and these intervals would be great value to improve valuable leaf type in future soybean breeding.

## Methods

### Plant materials and field trials

A GB RIL population with 256 RILs was obtained from a cross between ‘Guizao 1’ (ovule parent) and ‘Brazil 13’ (pollen parent) using a single seed descent (SSD) method derived from individual F_2_ plants [72]. Guizao 1’ is a cultivar from Cash Crops Research Institute, Guangxi Academy of Agricultural Sciences. ‘Brazil 13’ was introduced from a Brazilian germplasm variety named ‘BRSMG 68’. Another ZH RIL population, which contains 164 RILs was also developed with SSD approach from ‘Zhonghuang 24’ (female parent) and ‘Huaxia 3’ (male parent). ‘Zhonghuang 24’ is a cultivar adaptive to Huang-Huai-Hai Rivers Valley China. ‘Huaxia 3’ is a high-yielding variety which, and was obtained from by South China Agriculture University. The F_13_ GB RILs together with both parents were planted at the Zengcheng experimental station (N23°24′, E113°64′) in the summer of 2016 for the pre-experiment; the F_14_ GB RILs were grown at the Zengcheng experimental station in the spring of 2017 and in the summer of 2017 both at the Zengcheng experimental station and the Guangzhou experimental station (N23°15′, E113°34′). The F_12_ ZH RILs were grown together with both parents at the Zengcheng experimental station in the spring and summer of 2017. We adopted a randomized complete block planting with three replications. Each plot contained 10 plants per row, with 0.5 m between the rows and 0.1 m between the plants. Field management followed normal soybean production practices for the area.

### Measurement of leaf type traits and data analysis

The testing targets were the five plants in the middle of each row. According to the former research carried out by Hanway and Thompson, we concentrated on four representative growth stages for leaf type associated traits scoring: the 6th node vegetative stage (V6), the full bloom reproductive growth stage (R2), the full pod reproductive growth stage (R4), and the full seed reproductive growth stage (R6) [73]. The material testing condition details are as listed below: ZH RIL population, the V6, R4 and R6 growth stages in the summer of 2017 at Zengcheng, the R2 growth stage in the spring of 2017 at Zengcheng; GB RIL population, the V6 growth stage in the summer of 2017 at Zengcheng, the R2 growth stage in the spring of 2017 at Zengcheng, the R4 growth stage in the summer of 2016 at Zengcheng, the R6 growth stage in the summer of 2017 at Zengcheng and Guangzhou two experimental stations.

We took the fully developed middle leaflets with petioles of the third node on the main stem counting down from the top. The leaf samples were collected and stored in a 4°C room and waited for the test [9]. By using an EPSON scanner, Picasa 3 (https://picasa.en.softonic.com/) and Image-Pro Plus 7.0 (http://www.mediacy.com/) software, we obtained the phenotypic values for LA, LL, LW and PL. The LS values were determined by the ratio LL to LW. Frequency distribution graphs were created by Graphpad prism 7.0 (http://www.graphpad.com/). Statistical analysis was calculated by SPSS Statistics 19.0 (https://www.ibm.com/products/spss-statistics).

### Genetic map and QTL detection

#### SNP genotyping

Using a SOAP aligner (http://soap.genomics.org.cn/) software, the sequencing reads of the parents and each RILs were aligned to the soybean reference genome from Williams 82 [74]. The SOAP alignment results were formatted and then converted into input files using SAMtools (http://samtools.sourceforge.net/) [75]. The SNPs in RILs were identified by realSFS software. The likelihoods of genotypes for each individual were integrated and extracted as candidate SNPs and then filtering these SNPs by following criteria: 40 ≤ depth ≤ 2500, sites with a probability ≥95%. Adopting the sliding window approach which contained 15 SNPs per window, to identify the genotype for each window and the exchange sites for each individual by sliding an SNP every time. Finally, the genotypes for each individual were applied for generating bin information [76]. All the genotyping work was conducted at the Beijing Genome Institute (BGI) Tech, Shenzhen, China.

#### QTL detection

Based on 0.2 × RAD-seq and the bin genotypes of the RIL populations, two high-density genetic linkage maps were ultimately constructed by MSTMap (http://alumni.cs.ucr.edu/~yonghui/mstmap.html) and MapChart (https://www.wur.nl/en/show/MapChart-2.32.htm) [77]. The composite interval mapping (CIM) method was employed to scan QTLs by WinQTLCart (http://statgen.ncsu.edu/qtlcart/WQTLCart.htm) software [78, 79]. The LOD thresholds for QTL significance were evaluated by 1000 replications test with a genome-wide at the 5% level of significance to justify the existence of QTLs. According to the tests, a LOD score of 2.5 was used as a minimum to announce the presence of a QTL in a particular genomic region [80]. Running result of software can show additive effects of QTLs and phenotypic variation. QTL mapping results were comprehensively compared to SoybaseDatabase (https://www.soybase.org/).

#### QTL naming

According to Cui et al., all the QTLs were named as follows: initial ‘q’ denotes ‘QTL’; following with leaf type associated traits abbreviation letters; the next number is the soybean chromosomes on which the QTL is distributed [81]. Moreover, letters ‘a’ to ‘d’ represent the QTL was detected in ZH RIL population at the V6, R2, R4 and R6 growth stages at Zengcheng, respectively; letters ‘e’ to ‘i’ refer to the QTL for GB RIL population detected at the V6, R2, R4 and R6 growth stages at Zengcheng, respectively; letter ‘i’ means the QTL was discovered in GB RIL population at the R6 growth stage at the Guangzhou experimental station; if more than one QTL for a specific leaf trait was dispersed along a certain chromosome, a serial number, viz.-1, 2, etc., is used after the ‘a’ to ‘i’ to describe their order.

#### Gene ontology (GO) enrichment analysis

In this study, the Glyma.Wm82. a1. v1.1 gene model from SoyBaseDatabase was used for identification of the genes that fall into the genetic intervals of the detected QTLs. The AgriGo toolkit v2.0 (http://systemsbiology.cau.edu.cn/agriGOv2) was utilized to perform gene ontology (GO) analysis for these genes [82].

## Additional file

Additional file 1: **Table S1.** The pairwise correlation coefficients between different leaf type traits in ZH RILs across multi-environments. **Table S2.** The pairwise correlation coefficients between different leaf type traits in GB RILs across multi-environments. **Table S3.** Correlation coefficients between different environments for leaf type traits in ZH RILs. **Table S4.** Correlation coefficients between different environments for leaf type traits in GB RILs. **Table S5.** Description of characteristics of 20 chromosomes in ZH RIL population high-density genetic map. **Table S6.** Description of characteristics of 20 chromosomes in GB RIL population high-density genetic map. **Table S7.** Fifty-six QTLs and 48 loci for leaf type traits in ZH RIL population across environments. **Table S8.** One hundred thirty-four QTLs and 100 loci for leaf type traits in GB RIL population across environments. **Table S9.** Eleven QTL hotspots associated with leaf type traits detected in ZH RIL population. **Table S10.** Twenty-three QTL hotspots associated with leaf type traits detected in GB RIL population. **Table S11.** Gene models on Genome build Glyma 1.1 Chromosome of four important QTL hotspots. **Table S12.** Gene Ontology (GO) enrichment analysis of four important QTL hotspots. **Table S13.** Gene models on Genome build Glyma 1.1 Chromosome of specific leaf type traits. **Table S14.** Gene Ontology (GO) enrichment analysis of specific leaf type traits. **Table S15.** Information of SoybaseDatabse published leaf type traits associated QTLs. **Table S16.** Comparisons of the dected QTLs in ZH RIL population between present research and previous stuides. **Table S17.**Comparisons of the dected QTLs in GB RIL population between present research and previous stuides. **Table S18.** Four important QTL hotspots contain several published leaf type and yield-related traits QTLs. (XLS 428 kb)

## Abbreviations

bp: Base pair
CIM: Composite interval mapping
cM: Centimorgan
cv.: cultivar
GO: Gene ontology
LA: Leaf area
LAI: Leaf area index
LL: Leaf length
LS: Leaf shape
LW: Leaf width
MAS: Molecular assisted selection
NGS: Next generation sequencing
PL: Petiole length
QTL: Quantitative trait locus
R^2^: Phenotypic variance
RAD-seq: Restriction-site associated DNA sequencing
RIL: Recombinant inbred line
SAM: Shoot apical meristem
SNP: Single nucleotide polymorphism
SSD: Single seed descent
